# Challenges in Anaesthesia Management of a 15-Year-Old Female With Ovarian Teratoma for Exploratory Laparotomy: A Case Report

**DOI:** 10.7759/cureus.29175

**Published:** 2022-09-14

**Authors:** Sambit Dash, Sanjot Ninave, Amol Bele, Haneesha Movva, Manish Sonkusale

**Affiliations:** 1 Department of Anaesthesiology, Jawaharlal Nehru Medical College, Datta Meghe Institute of Medical Sciences (Deemed University), Wardha, IND; 2 Department of Anaesthesiology, Acharya Vinoba Bhave Rural Hospital, Datta Meghe Institute of Medical Sciences (Deemed University), Wardha, IND

**Keywords:** general anaesthesia, re-expansion pulmonary oedema, respiratory failure, debulking surgery, exploratory laparotomy, growing teratoma syndrome, ovarian teratoma

## Abstract

Rarely, an ovarian tumour will develop the growing teratoma syndrome. Growing teratoma syndrome of the cystic type has been linked to difficulties with anaesthesia because of the abdominal pressure the tumour exerts on the thorax. There haven't been any reports of this kind of ovarian tumour associated with ascites and bilateral pleural effusion in a paediatric age group. Here, we describe our anaesthetic experience in a case of developing solid-type ovarian teratoma syndrome with deranged lung status and haemodynamics.

The patient was a 15-year-old female who was diagnosed with ovarian teratoma. She was scheduled for surgery when she arrived at our hospital with a 13 cm solid mass and respiratory distress. The patient's liver profile was abnormal; she had ascites, pleural effusion and a severely worsened lung condition. The patient was planned for an exploratory laparotomy and debulking surgery after preoperative optimisation. To prevent the re-expansion pulmonary oedema (RPO) following the excision of the tumour, a volume-restricted postoperative ventilation strategy was planned. Following enhanced recovery after surgery (ERAS) protocol and specific anaesthetic measures, we successfully managed the anaesthesia in a case of teratoma syndrome with a large abdominal tumour with successful recovery and early discharge from hospital.

## Introduction

It is current practice to consider patients up to the age of 18 to be paediatric [1]. Gynaecological oncology includes ovarian cancers in young children and adolescent females as a significant component. One percent of all childhood cancers and 8% of paediatric abdominal tumours are of ovarian origin and are the most prevalent gynaecological neoplasm detected in female children. Additionally, 10%-30% of ovarian neoplasms removed from young or adolescent females are malignant [2].

In most cases, it is difficult to decide on the most effective surgical treatment, which puts gynaecologists and surgeons caring for these patients in a dilemma. This also puts the pathologist in a challenging scenario when making a diagnosis because these two aspects have an impact on the likelihood of recurrence and the prospect of future childbearing.

It is generally known that in the first two decades of life, germ cell tumours are the most prevalent ovarian neoplasm, making up roughly two-thirds of all ovarian cancers. One-third of germ cell origin cancers and two-thirds of all ovarian malignancy in this age group are malignant germ cell tumours [3,4].

The prognosis of malignant ovarian germ cell tumours has improved recently compared to previous decades due to significant management-related advances, but it still requires accurate histological diagnosis with staging, tumour marker estimation and immunohistochemistry, when necessary, as well as patient motivation through appropriate counselling to increase survival rates [5].

## Case presentation

A 15-year-old female presented to the gynaecology outpatient department (OPD) with a complaint of intermittent pain in the abdomen associated with abdominal distention and decreased appetite over the preceding 8-10 days. She complained of a dull aching umbilical and epigastric pain that was not relieved by any medications. There was no complain of breathlessness, chest pain, nausea, vomiting, weight loss, bleeding per the vagina or white discharge. The patient's vitals in OPD were as follows: heart rate (HR): 90/minute, blood pressure (BP): 118/78 mmHg, oxygen saturation (SpO_2_): 98% on room air and respiratory rate (RR): 20 breaths/minute. Abdominal ultrasound was done. They reported a solid cystic mass measuring 13.3 cm × 10.2 cm × 9 cm suggestive of ovarian teratoma, with moderate ascites. Hence, later, the patient was admitted in gynaecological ward.

**Figure 1:**
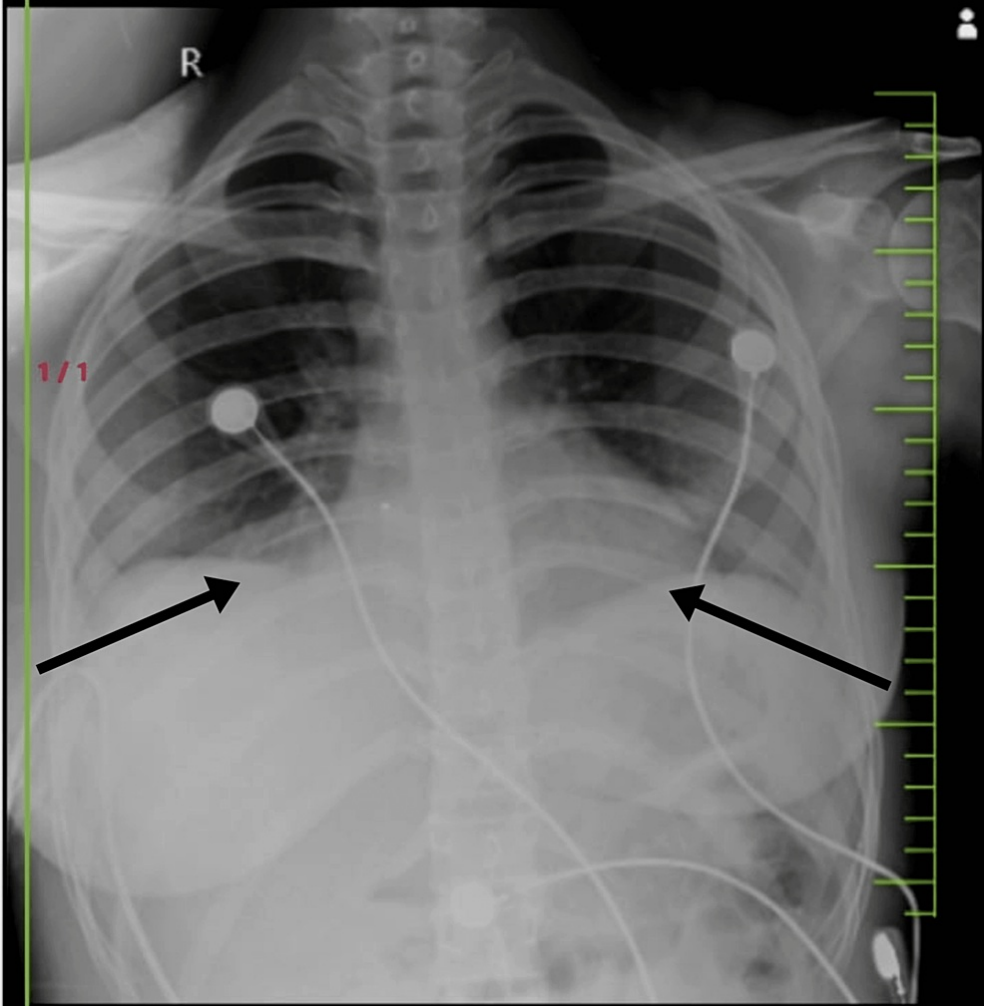
Chest X-ray on day 2 of admission. We can see that the costophrenic angles on bilateral lungs are obliterated. The chest X-ray indicates mild bilateral pleural effusion. The X-ray was advised after the patient started complaining of dyspnoea owing to her growing ovarian teratoma.

On day 2 of admission, the patient complained of dyspnoea. So, chest X-ray (Figure 1) and ultrasonography (USG) thorax were done. USG thorax revealed bilateral pleural effusion (right > left) with subsegmental atelectasis. USG thorax was followed by high-resolution computed tomography (HRCT) thorax for a more definitive finding. It revealed right moderate and left mild pleural effusion with atelectasis with minimal subpleural atelectasis. SpO_2_ was 98% on room air. Ascitic tapping was done the same day, and 950-1,000 ml of fluid was drained. It was followed by pleural tapping, and around 300 ml of pleural fluid was also tapped from the right side.

**Figure 2:**
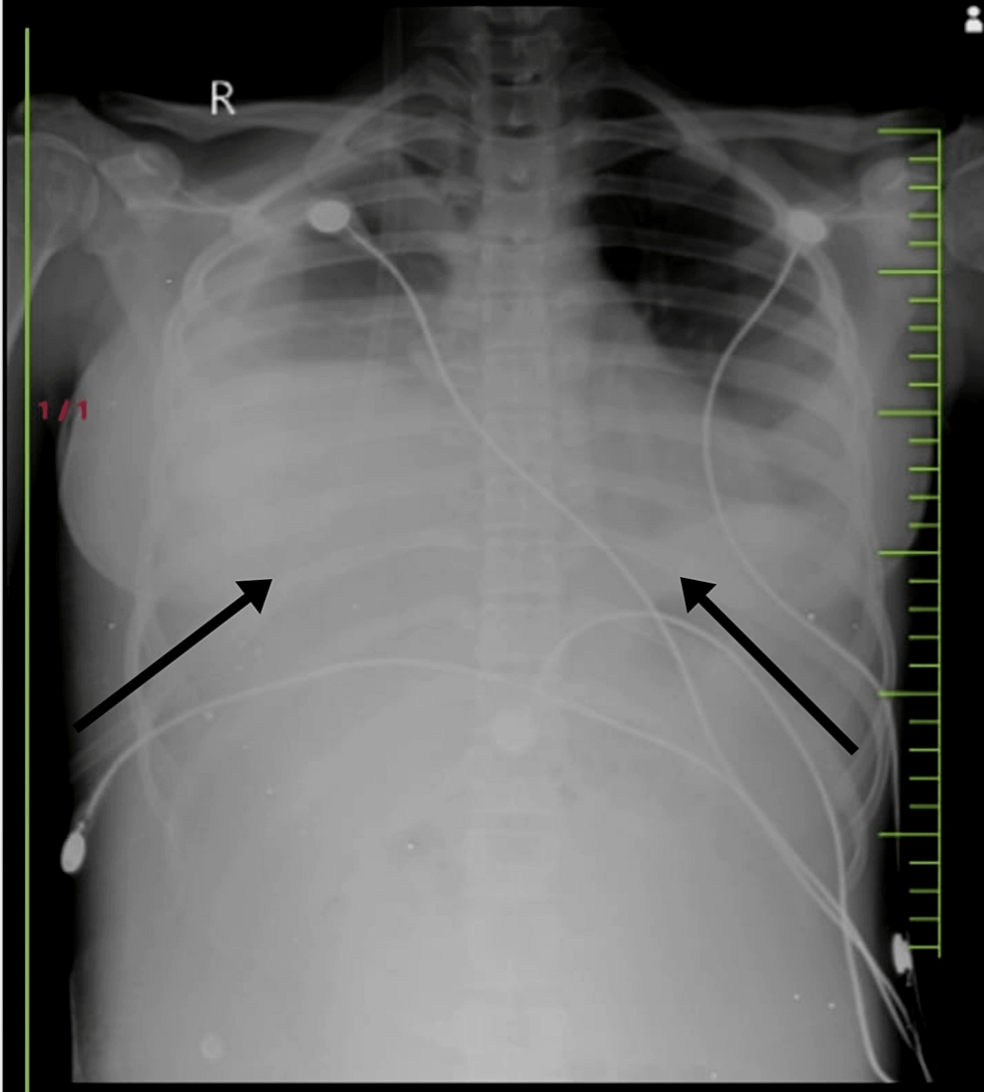
Chest X-ray on day 4. The chest X-ray shows significant obliteration of the bilateral costophrenic angles. It indicates moderate pleural effusion. The X-ray was advised when the patient suddenly desaturated to 89% on room air and started complaining of breathlessness and dyspnoea.

**Figure 3:**
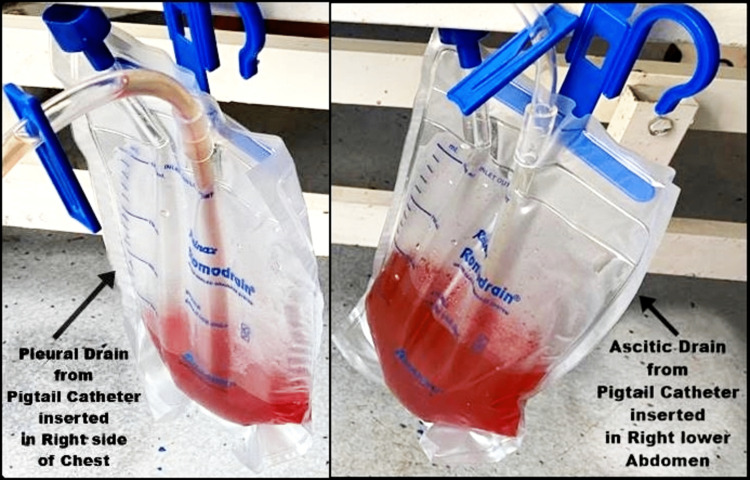
Picture of pleural and abdominal drain showing collections. Two pigtail catheters were put in situ by an interventional radiologist, one on the right side of the chest and the other on the right lower abdomen. The decision to secure a pigtail catheter was taken owing to the repeated pleural and ascitic fluid collection and multiple ascitic and pleural tapping.

On day 4, she developed sudden respiratory distress, and peripheral oxygen saturation was 89% on room air. Hence, the patient was shifted to medicine ICU for further management, and bedside chest X-ray was done (Figure 2). Chest X-ray showed moderate bilateral pleural effusion (right > left). Hence, percutaneous pigtail catheter was inserted and secured on the right side of the chest and right lower abdomen by interventional radiologist. Per day drain of ascitic and pleural fluid was around 300-400 ml each (Figure 3). The patient developed bilateral pitting pedal oedema after two days, so liver function test (LFT) was repeated, which showed hypoalbuminemia. Serum albumin levels decreased from 3.6 gm/dl to 1.8 gm/dl. Injection albumin 20% 100 ml per day solution was transfused for three days (Table 1).

**Table 1:** Liver function test done on day 6 after the patient developed bilateral pitting oedema and myalgia owing to massive drain of ascitic and pleural fluid. It shows significant hypoalbuminemia with serum albumin level at 1.8 and a reversed albumin:globulin ratio. SGPT: serum glutamic pyruvic transaminase; SGOT: serum glutamic oxaloacetic transaminase; ALP: alkaline phosphatase; T: total.

SGPT	SGOT	ALP	T bilirubin	Conjugated	Unconjugated	T protein	Albumin	Globulin
78	82	180	1.4	0.8	0.6	4.0	1.8	2.2

Gradually, the pedal oedema subsided, and the albumin levels improved to 2.8 gm/dl along with a decrease in volume of ascitic and pleural fluid collection in the drain also over a period of two weeks. Now, debulking surgery and exploratory laparotomy were planned. During pre-anaesthetic checkup (PAC), the patient was febrile with temperature of 101.8 degrees Fahrenheit. On auscultation, air entry was significantly reduced in the right axillary, right interscapular area and bilateral lower lobes, owing to the increased intra-abdominal pressure due to ascites and tumour mass. The pleural fluid collected in the drain was haemorrhagic and approximately 125 ml. SpO_2_ was 95% on room air. Breath holding time was 18 seconds, and single breath count was 16 while doing bedside pulmonary function test (PFT). The height and weight of the patient were noted to be 151 cm and 44 kg, respectively.

The patient was given provisional fitness if afebrile on the day of surgery under high risk with surgical intensive care unit (SICU) bed and postoperative ventilator support reserved and blood and blood products arranged. She was prescribed nebulisation three to four times a day with salbutamol and Budecort and asked to repeat chest X-ray on the day of surgery along with temperature charting. Plan of anaesthesia was epidural with general anaesthesia. Enhanced recovery after surgery (ERAS) protocol was followed for the patient. Proper preoperative counselling was done; adequate preoperative nutrition was maintained. The patient was kept nil by mouth for six hours preoperative, with adequate maintenance of intravenous (IV) fluids.

On the day of surgery, fresh chest X-ray showed obliteration of bilateral costophrenic angles suggestive of bilateral pleural effusion (moderate on the right side and minimal on the left). On auscultation, air entry was decreased in bilateral lower lobes. After explaining high-risk consent to parents, the patient was shifted on operation theatre (OT) table. Baseline HR was 100, non-invasive blood pressure (NIBP) was 110/78 mmHg, SpO_2_ was 95% on room air and ECG was normal. Two peripheral IV lines with 18 G intracath was secured on either hands. Under all aseptic precautions, 18 G epidural catheter was secured at L3-L4 vertebral interspace. The patient was premedicated with injection pantoprazole 40 mg, injection glycopyrrolate 0.2 mg, midazolam 1 mg and fentanyl 50 mcg. The patient was induced with injection propofol 80 mg and injection atracurium 20 mg, and the airway was secured with 7 mm endotracheal tube and maintained on oxygen, air (2 L each) and sevoflurane and titrated according to the blood pressure. Top-up dose of injection atracurium 5 mg was administered based on train of four count. Tidal volume (TV) was kept approximately 6 ml/kg at 275 ml.

**Figure 4:**
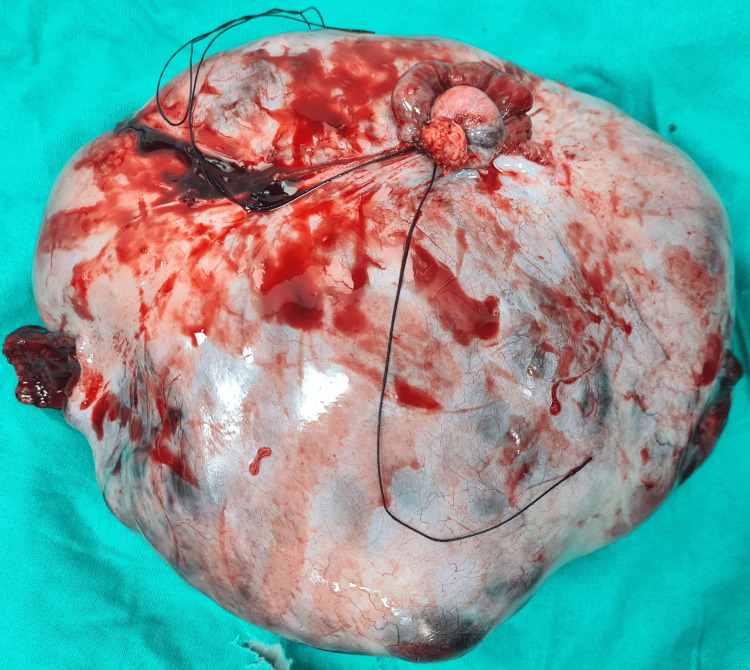
Image showing specimen. On measuring, it was found to be 13.3 cm × 10.2 cm × 9 cm in size. This growing ovarian teratoma led to the growing teratoma syndrome causing massive pleural effusion and ascites and compressing the adjoining major vessels leading to haemodynamic imbalance.

**Figure 5:**
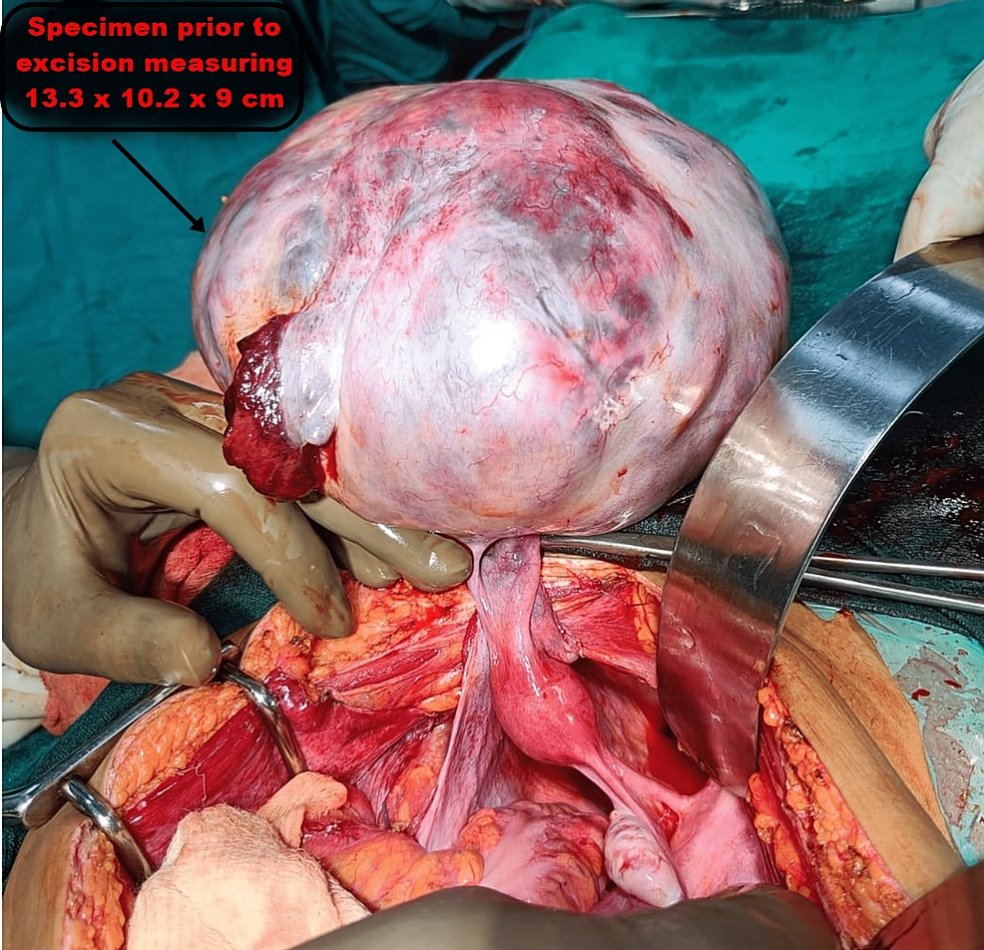
Intraoperative image showing the giant abdominal tumour prior to its excision.

A ultrasound-guided 7 Fr triple lumen central venous catheter was secured in the right internal jugular vein, and ultrasound-guided 20 G peripheral catheter was placed in the right radial artery for invasive blood pressure monitoring. Epidural catheter was tested with 3 ml of injection lignocaine 2% + adrenaline, and later, 8 ml of 0.25% bupivacaine was injected slowly while monitoring the haemodynamics and repeated every two hourly. Intraoperatively, about 1,000 ml of ascitic fluid was collected after opening the abdomen. Injection albumin 20% 100 ml was administered. Blood loss was estimated to be around 600 ml (Figures 4, 5). Haemodynamics were maintained throughout by adequate and timely replacement of losses with calculated amount of fluid and blood products. Intraoperatively, 150 ml of packed red blood cells (PRBC) was slowly transfused to the patient while constantly monitoring the urine output and the vitals of the patient. The total urine output during the five-hour surgery was around 400 ml. The chest was auscultated at regular interval to look for any crepitations or added sounds, and random blood sugar (RBS) was monitored every hourly. Blood glucose level was maintained between 100 and 120 mg/dl. Towards the end of surgery, arterial blood gas (ABG) done demonstrated mild metabolic acidosis compensated by respiratory alkalosis. Therefore, tidal volume and respiratory rate were decreased accordingly. The remaining 150 ml of blood was transfused postoperatively.

Postoperatively, the patient was transferred to paediatric ICU with endotracheal tube in situ and was maintained on injection midazolam infusion at 2 mg/hour until the coming morning of postoperative day (POD) 1. Ventilator setting was gradually weaned off, and the patient was allowed to take spontaneous breaths the next morning. After successful spontaneous respiration trial, the patient was put on T-piece with oxygen support. On the evening of POD 1, the patient was extubated successfully and maintained SpO_2_ on room air. In POD 3, the patient was shifted to paediatric ward for further management and later discharged on POD 12.

## Discussion

For our patient, ERAS protocol was adhered to. Enhance recovery programmes (ERPs), sometimes known as 'fast-track' programmes or ERAS, were developed by Kehlet [6] and became a significant part of postoperative care. These initiatives aim to alter the physiological and psychological reactions after major surgery, and it has been demonstrated that they reduce complications and length of hospital stay, improve cardiopulmonary function, hasten the return of bowel function and hasten the return to normal activities. The ERAS protocol's main tenets are preoperative counselling, preoperative nutrition, avoiding perioperative fasting, avoiding carbohydrate loading up to two hours before surgery, standard anaesthetic and analgesic regimens (epidural and non-opioid analgesia) and early mobilization [7].

General anaesthesia and anaesthetic care for tumour removal in cases of ovarian teratoma carry a number of complications when a large abdominal tumour is present [8]. Management of the circulatory and respiratory systems is particularly challenging. The four key issues in anaesthesia management are (1) the impact of tumour pressure on the lungs and great vessels on respiration, (2) the risk of aspiration during intubation, (3) the risk of significant bleeding and (4) the incidence of re-expansion pulmonary oedema (RPO).

A further issue with giant abdominal tumours is the management of the ventilatory system following the administration of a muscle relaxant because of decreased lung and thoracic compliance brought on by the relaxation of the diaphragm and the enlarged abdomen, the potential for high airway pressures to result in lung injury and the decreased lung and thoracic compliance [9].

Therefore, it has been recommended that, even when muscle relaxants are used, spontaneous breathing should be continued for as long as feasible and that the inspiratory pressure be kept below 20 cm H20. After the muscle relaxant was administered, spontaneous breathing stopped, as was to be expected, and we were unable to create a suitable positive pressure ventilation environment. Tidal volume (TV) quickly improved after making an abdominal midline incision. We recommended this procedure for individuals with a massive abdominal tumour whose mass cannot be reduced preoperatively because our overall goal was to sustain spontaneous respiration for as long as possible.

In cases with ovarian teratoma, there is typically a large mass that is occasionally quite close to the major vessels, but thorough surgical excision is necessary to stop the condition from relapsing [10,11]. Consequently, there is a chance of significant bleeding. In our situation, the removal of the tumour resulted in a blood loss of more than 600 ml, and 150 ml of PRBC was transfused during the operation. However, due to preoperative cannulation (central venous catheterisation and catheterisation of the radial artery for intra-aortic balloon pump (IABP) monitoring) and preparation for percutaneous cardiopulmonary support (PCPS), we were able to prevent blood loss and cardiac failure and maintain stable haemodynamics throughout the procedure.

After the tumour was removed, there was a danger of re-expansion pulmonary oedema (RPO) [12,13]. The duration of the chronic lung collapse serves as one of the risk factors for RPO, which manifests as fast expansion of chronically collapsed lungs due to increased pulmonary vascular permeability. RPO starts right away and frequently happens within an hour. Although there is no established way to stop RPO, in one instance, it was believed that keeping a TV between 6 and 8 ml/kg during surgery was to blame. It has also been argued that it is preferable to let collapsed lungs expand naturally over time rather than using a lung recruitment method to stop RPO [14].

In our case, the patient had poor pulmonary function prior to surgery, and the patient's lungs had been long since collapsed by the pressure of the enormous tumour. She was therefore a high-risk patient for RPO, and RPO could have developed during surgery. To avoid this from happening, we decided to very gradually expand the compressed lungs and kept the TV at a low level at around 5-6 ml/kg after the tumour was removed [15]. With this strategy, we were able to treat the patient without incident both during and after the procedure.

Additionally, the patient's liver function test revealed abnormal results due to the patient's severe hypoalbuminemia, which was brought under control before surgery by an intravenous albumin transfusion that increased the patient's serum albumin from 1.8 g/dl to 2.8 mg/dl. The metabolism of food and drugs, plasma protein synthesis, crucial haemostatic components, detoxification and the exclusion of several endogenous and foreign chemicals are only a few of the physiological systems whose equilibrium the liver contributes significantly to [16]. On the other hand, it has a role in host immunological responses to inflammation, sepsis and damage. Injection atracurium was the preferred muscle relaxant as a result of the deranged liver profile and low serum albumin level.

Patients with compensatory liver disease typically take inhalation anaesthetics, opioids and intravenous sedative-hypnotic drugs well. Because they may have long-lasting effects on consciousness and haemodynamics and lead to hepatic encephalopathy, they should be administered with caution in patients with decompensatory hepatic dysfunction.

Thoracic epidural anaesthesia (TEA) gives great pain relief during abdominal or thoracic procedures and may lower postoperative mortality [17]. Investigations have revealed that halothane should be avoided while administering general anaesthesia to these individuals since sustaining hepatic blood flow is crucial for patients with abnormal liver function test (LFT). Of all the inhalation anaesthetics, this one causes the most pronounced reduction in hepatic blood flow, oxygen supply and postoperative hepatic dysfunction. If an inhalational approach is chosen for these patients, isoflurane seems to be a preferable option. Sevoflurane may offer certain advantages over other volatile anaesthetics, according to more recent volatile anaesthetics such as desflurane and sevoflurane, even though there isn't much of a difference between them.

To draw firm conclusions and make decisions about these anaesthetic drugs, more research has been suggested. For many years, nitrous oxide has been administered without any complications to patients with severe liver disease. Because of its sympathomimetic effects, some people think that administering nitrous oxide in patients with severe liver disease could endanger oxygenation. On the other hand, prolonged nitrous oxide anaesthesia may cause gas to build up in the intestinal lumen and subsequently cause intestinal distension.

Patients with hepatic illness have successfully utilised opioids. However, certain pharmacological effects such as a prolonged half-life and a delayed drug clearance should be taken into account [18,19]. Given its ability to maintain hepatic blood flow and oxygen levels while being administered in relatively moderate doses, fentanyl is regarded as the opioid of choice in these patients.

The effect of medications such as d-tubocurarine and pancuronium, which are used as muscle relaxants, can be prolonged due to the fall in hepatic blood flow, hepatic metabolism and excretory functions, as well as compromised renal function. According to studies, vecuronium's pharmacokinetics are not considerably impacted by advanced liver disease. Because atracurium is metabolised by Hoffman's degradation and body temperature, it offers a theoretical benefit. Therefore, in individuals with compromised hepatic or renal function, clearance and elimination half-life of atracurium are comparable to those of patients with normal hepatorenal function.

## Conclusions

In conclusion, we've provided a description of how to successfully manage anaesthesia in a case of giant ovarian teratoma. Numerous hazards were involved in the onset and maintenance of anaesthesia, and timely and thoughtful transfusions were necessary for circulatory control. A 15-year-old female with a huge ovarian teratoma, bilateral pleural effusions and ascites was the patient in this instance. After the tumour was removed, respiratory control was accomplished by maintaining a relatively low TV, comparable to that during preoperative spontaneous respiration, in order to prevent re-expansion pulmonary oedema. There was severe hypoalbuminemia as a result of the pleural and ascitic fluid draining. Optimising the patient's blood profile and haemodynamics was a crucial aspect of preoperative care and the pre-anaesthetic checkup.

Intraoperatively, blood and fluid loss was well anticipated, and central venous access was taken, and invasive blood pressure monitoring was done. Timely replacement of blood and fluid loss was given after close monitoring of input versus output. As a result, haemodynamics was successfully maintained throughout. Postoperatively, the patient was shifted with endotracheal tube in situ and electively ventilated until the next day morning owing to poor lung status preoperatively. Proper preoperative assessment followed by management coupled with judicious intraoperative planning and execution led to successful anaesthetic management of exploratory laparotomy in a paediatric female with massive growing teratoma, which otherwise can become a perioperative challenge for any anaesthesiologist.
